# Mathematical model of the cell signaling pathway based on the extended Boolean network model with a stochastic process

**DOI:** 10.1186/s12859-022-05077-z

**Published:** 2022-11-30

**Authors:** Minsoo Kim, Eunjung Kim

**Affiliations:** Natural Product Informatics Research Center, Korea Institute of Science and Technology, Gangneung, South Korea

**Keywords:** Extended Boolean network model, Stochastic process, MAPK signaling pathway

## Abstract

**Background:**

In cell signaling pathways, proteins interact with each other to determine cell fate in response to either cell-extrinsic (micro-environmental) or intrinsic cues. One of the well-studied pathways, the mitogen-activated protein kinase (MAPK) signaling pathway, regulates cell processes such as differentiation, proliferation, apoptosis, and survival in response to various micro-environmental stimuli in eukaryotes. Upon micro-environmental stimulus, receptors on the cell membrane become activated. Activated receptors initiate a cascade of protein activation in the MAPK pathway. This activation involves protein binding, creating scaffold proteins, which are known to facilitate effective MAPK signaling transduction.

**Results:**

This paper presents a novel mathematical model of a cell signaling pathway coordinated by protein scaffolding. The model is based on the extended Boolean network approach with stochastic processes. Protein production or decay in a cell was modeled considering the stochastic process, whereas the protein–protein interactions were modeled based on the extended Boolean network approach. Our model fills a gap in the binary set applied to previous models. The model simultaneously considers the stochastic process directly. Using the model, we simulated a simplified mitogen-activated protein kinase (MAPK) signaling pathway upon stimulation of both a single receptor at the initial time and multiple receptors at several time points. Our simulations showed that the signal is amplified as it travels down to the pathway from the receptor, generating substantially amplified downstream ERK activity. The noise generated by the stochastic process of protein self-activity in the model was also amplified as the signaling propagated through the pathway.

**Conclusions:**

The signaling transduction in a simplified MAPK signaling pathway could be explained by a mathematical model based on the extended Boolean network model with a stochastic process. The model simulations demonstrated signaling amplifications when it travels downstream, which was already observed in experimental settings. We also highlight the importance of stochastic activity in regulating protein inactivation.

**Supplementary Information:**

The online version contains supplementary material available at 10.1186/s12859-022-05077-z.

## Background

Since Stuart Kauffman introduced the gene regulatory network concept [1] nearly five decades ago, the Boolean network model approach has been extensively utilized to study complex signaling pathways, such as modeling the cell differentiation process [2]. Dynamic analysis of mammalian cell-cycle networks using the Boolean model is also reported [3]. The classical mechanism of G_protein-mediated signaling was demonstrated based on a pattern-matching approach to associate gene expression profiles with the Boolean model [4]. In addition, the Boolean modeling approach is often integrated with multiple perturbations for phosphoproteome time series [5] and drug response data using a microfluidics perturbation screening strategy to develop cell line- and patient-specific logic models [6]. Recently, a Boolean model with a stochastic update algorithm was proposed to describe and predict the signaling network for abscisic acid (ABA)-induced stomatal closure. The developed model successfully described ABA sensitivity and accurately captured the effect of knock-out and constitute activity, and most of the simulation results were in good agreement with the experimental data [7]. In addition, a Boolean model of prostate cancer signaling pathways successfully differentiated healthy cells from cancerous cells. By integrating with patient data, the model classified patient Gleason score grade for each patient [8].

The Boolean network model applied to the previous model assumes a binary set of protein states [9]. It is often difficult to describe a more complex interaction with the modeling approach since the interaction between two proteins can be more complex than a binary set (TRUE or FALSE). On the other hand, using weights representing the strength of the interaction between two proteins can allow for more flexibility and complexity in the dynamic interaction. To this end, the extended Boolean model was developed to include the weights so that the activity of the network can be expressed as a continuous variable (typically in [0, 1]). For example, a fuzzy set operator calculates the Boolean logic operators “AND”, and “OR” as the maximum or minimum for the weights [10]. Briefly, a fuzzy set is defined as a mapping from a set to a Boolean lattice. Fuzzy logic is a multi-valued logical structure and uses a sequence of logical values between 0 and 1, which is completely FALSE or TRUE, respectively. The fuzzy set operator has a single operand dependency problem due to the MAX and MIN operators. Thus, it is difficult to calculate the Boolean value of weights simultaneously through the entire evaluation mechanism, especially when the evaluation function has the associative property.

The Waller–Kraft operator was introduced as an extension of the fuzzy set operator, which utilizes a linear combination for the maximum and minimum of weights [11]. The *p-norm* operator is based on the concept of Euclidean distance, whereas Boolean operators are used for all weights [12]. The operator offers the advantages of a short calculation time and utilizes the maximum and minimum weights simultaneously over the fuzzy set operator. The operator can also control a coefficient of logic operators (“AND”, “OR”) using a user-specified parameter. A stochastic process was also integrated into the Boolean network model by shifting the homogeneous Poisson point process along the stochastic process to maintain the Boolean model with the original distribution at each fixed time *t* [13]. Moreover, an efficient probabilistic Boolean network modeling approach was developed for the p53-MDM2 network and the T cell immune response [9].

This study presents a new mathematical model of cell signaling pathways based on the extended Boolean method with the Waller–Kraft operator and a stochastic process. The model was employed to simulate the mitogen-activated protein kinase (MAPK) signaling pathway. The MAPK signaling pathway is one of the most well-studied pathways [14–16], which regulates critical cellular processes such as differentiation, proliferation, apoptosis, and survival in response to various micro-environmental stimuli in eukaryotes (reviewed in [17, 18]). Upon receiving a micro-environmental stimulus, receptors on the cell membrane, such as the epidermal growth factor receptor (EGFR) and mesenchymal-epithelial transition (MET) receptor, become activated. Activated receptors then initiate a cascade of protein activation of downstream proteins in the MAPK pathway, which involves protein binding, thereby creating scaffold proteins [19] that are known to facilitate effective MAPK activation [20, 21]. The scaffold proteins serve as a platform for a single protein to assemble, coordinate feedback signals, and protect activated proteins (reviewed in [22, 23]). In the model, we assume that the activity of proteins in the pathway is regulated by a Boolean function, which is determined by the weights of protein–protein interactions. The model also considers the effect of stochastic factors of protein self-activity on signaling transduction. Cell signaling is affected by intrinsic and extrinsic stochastic factors [24]. Extrinsic stochasticity can be associated with inter-cellular fluctuation due to extracellular microenvironment changes. Intrinsic stochasticity can be associated with several factors, such as the Brownian motion of proteins in the cytoplasm of the cells [25], stochastic protein degradation [26], and self-activation of proteins [27]. In the model, these factors were represented by a stochastic process, following a normal distribution with a mean 0 and standard deviation *simulation time step*.

The developed model was employed to simulate a simplified MAPK signaling pathway activity in response to a single micro-environmental stimulus. We then compared the effect of stochastic factors on the signaling pathway. We also simulated the pathway activity in response to repeated multiple micro-environmental stimuli. Our results suggest that the extended Boolean network model can effectively simulate the MAPK pathway in response to different micro-environmental stimuli and further highlight the importance of a stochastic process for proper inactivation after the signaling pathway stimulation.

## Method

### Mathematical model assumptions

Our model describes changes in protein activity when the amount of intracellular protein is sufficient for activation to occur. The MAPK signaling is regulated by proteins that can simultaneously bind to multiple proteins in the pathway [21], creating a scaffold protein. The scaffold protein can help localize molecules in specific parts of the cell or improve the effectiveness of the signaling pathway [22], and further regulates the selectivity of the pathway to achieving new responses from the signaling components [23]. Thus, we considered that a scaffold could control the activity of each protein in the pathway. Furthermore, we included a weight between two proteins to represent the strength of protein–protein interactions, which may represent protein abundance or protein binding strength at each time in a cell. To formulate our mathematical model, we made the following additional assumptions:

1. Activated proteins can instantaneously bind to each other to create a scaffold for their immediate downstream protein activation.
2. Each protein binds to the scaffold independently from one another.
3. After interacting with the immediate downstream of an individual protein, the protein is released from the scaffold and becomes inactivated.
4. The strength (weight) of the interaction between proteins is randomly assigned with the uniform distribution [0, 1].
5. The weights do not change over time.

Assumptions (1) and (3) imply that the activity of each protein is regulated by binding and detaching from a scaffold. We set these assumptions based on experimental studies that investigated the role of a scaffold protein such as kinase suppressor of RAS (KSR) or STE5 in MAPK pathway signaling pathway [20, 28, 29]. It has been observed that the phosphorylation of KSR results in the release of protein in the MAPK pathway, such as RAF, from the scaffold complex, which in turn inactivates MEK [29]. We further assume that the physical binding and detaching from a scaffold are instantaneous. We do not model the direct physical process of binding or detaching. Assumption (2) indicates that an individual protein can bind to a scaffold by itself upon activation. We set assumption (4) to represent the different strengths of each protein interaction. Of note, we simulated 1000 simulations with different weights and reported an average behavior. Finally, for simplicity, we assume that the interaction weight does not change with time (assumption (5)).

### Mathematical model formulation

**Fig. 1:**
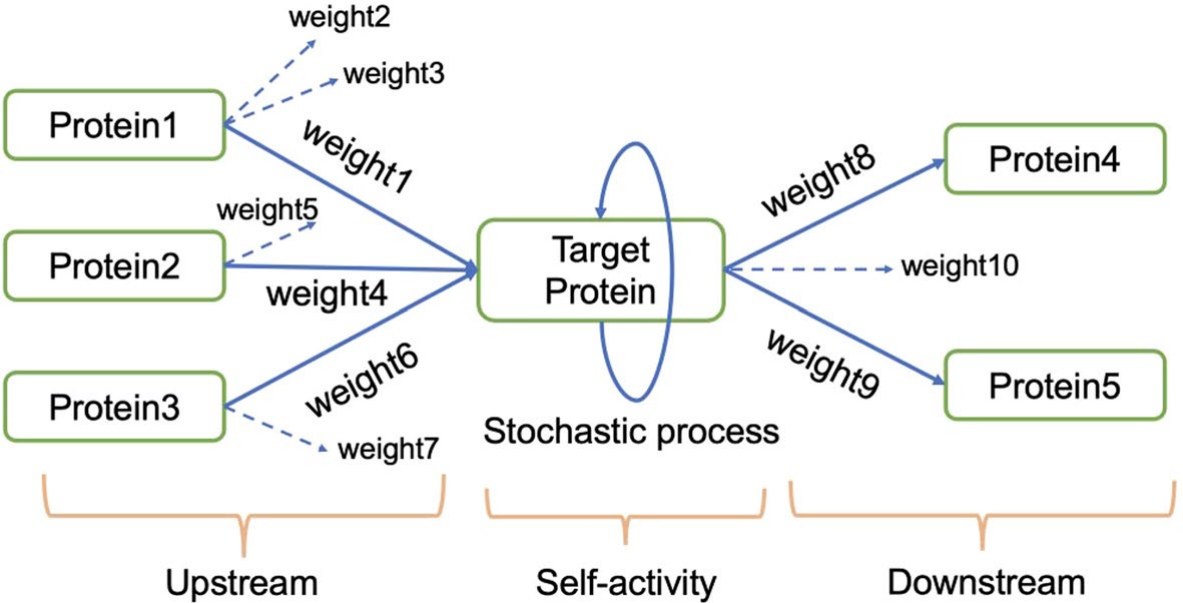
Cell signaling network diagram for model development explanation. For a target protein, the proteins 1–3 are referred to as upstream, which can activate the target protein with interaction strength weight 1, weight 4, and weight 6. The target protein can activate proteins 4 and 5 with interaction strength weights 8 and 9, respectively. The solid lines indicate interactions with the target protein. The interaction with other proteins is represented by dotted lines

To explain the model development process, we consider an example network composed of one target protein, three upstream proteins, and two downstream proteins (Fig. 1). In the network, the activation of the target protein is modulated by three factors; interactions with proteins in the upstream proteins, an inactivation after interaction with downstream proteins, and self-activation (stochastic production or degradation of a protein), as follows:

$$\begin{aligned} \text {activity of a target protein} = \text {upstream} - \text {downstream} + \text {self-activity}. \end{aligned}$$

Activation of the target protein by upstream proteins is calculated in three steps. First, the Boolean function is applied to all weights between the target protein and each protein in its immediate upstream protein to calculate the interaction strength between the two. Second, the influence of each protein on the target is calculated by multiplying the interaction strength with the activity of the upstream protein. Third, all of these influences are combined. For example, the effect of the upstream proteins on the target protein in Fig. 1 is calculated by summing the products of the Boolean function value of weights and each protein activity (see the equation below). Similarly, the activity of downstream proteins is modeled as a product of the activity of the target protein with the Boolean function value between the target and a downstream protein. The self-activation or inactivation of the target protein is modeled as a product of the target protein and a random variable that is normally distributed with mean zero and standard deviation *time step* ($\Delta t$).

$$\begin{aligned} {\text{upstream:}} & \;x_{1} \times B(w_{1} ,w_{2} ,w_{3} ) + x_{2} \times B(w_{4} ,w_{5} ) + x_{3} \times B(w_{6} ,w_{7} ), \\ {\text{downstream:}} & \;x_{T} \times B(w_{8} ,w_{9} ,w_{{10}} ), \\ {\text{self - activity:}} & \;x_{T} \times W, \\ \end{aligned}$$

where ${x_i}={\text {activity of protein}_i}$, ${x_{T}}={\text {activity of target protein}}$, $w=\text {weight}$, *W*= normally distributed random variable with mean 0 and standard deviation $\Delta t$, and $B=\text {Boolean function}$.

Accordingly, the activity of the target protein is expressed as

$$\begin{aligned} \displaystyle ({x_{1}}B(w_{1}, w_{2}, w_{3})+{x_{2}}B(w_{4}, w_{5})+{x_{3}}B(w_{6}, w_{7})) - ({x_{T}}B(w_{8}, w_{9}, w_{10})) + ({x_{T}}W). \end{aligned}$$

The Boolean values of the upstream and downstream proteins are calculated through a Boolean function (defined in **void** $\text {Boolean}\underline{}\underline{}\text {gate}$ in the Additional file 1). The stochastic process follows a normal distribution with mean 0 and standard deviation *dt* derived from the Euler-Maruyama method (described in the following section and defined as **double** $\text {gaussianRandom}$ in the Additional file 1).

The above protein activation process can be readily converted into a system of stochastic differential equations. We utilized the Waller–Kraft operator [11] dependent on the weights of each activity as the extended Boolean logic gate. In the model, we consider only “AND” logic to simulate simultaneously occurring protein binding and detaching. The parameter of the Waller–Kraft operator, *r*, can be chosen within [0.5, 1]. We constructed a model of a signaling pathway composed of *N* proteins as follows:

1 $$\begin{aligned}&dx_{i}(t)=\biggl (k_{u}\sum _{k\in n}B_{k}(w_{kj^{'}})x_k(t)-k_{d}B_{i}(w_{ij})x_{i}(t)\biggl )dt+\sigma x_{i}(t)dW(t), \end{aligned}$$

2 $$\sum\limits_{{k \in n}} {B_{k} } \left( {w_{{kj}}^{'} } \right)x_{k} (t) = \sum\limits_{{k \in n}} {\left( {r\min \left( {w_{{k1}} , \ldots ,w_{{km}}^{'} } \right) + \left( {1 - r} \right)\max \left( {w_{{k1}} , \ldots ,w_{{km}}^{'} } \right)x_{k} (t)} \right)} ,$$

3 $$\begin{aligned} B_{i}(w_{ij})=r\min (w_{i1},\ldots ,w_{im})+(1-r)\max (w_{i1},\ldots ,w_{im}), \end{aligned}$$

where $x_{i}(t)$ indicates the activity of each protein at time *t*, in which $1 \le i \le N$, *n* is the number of proteins in the upstream of $x_{i}$, and $x_{k}$ is an individual protein immediate upstream to $x_{i}$ so that $1\le k \le n$. $B_{k}$ is a Boolean function for the upstream interactions based on the Waller–Kraft operator. $j^{'}$ is the order of weights between $x_k$ and its upstream neighbors. $B_{i}$ is a Boolean function that acts downstream of $x_{i}$, and *j* is the order of weights between the protein $x_{i}$ and its downstream neighbors. Self-regulation of each target protein $x_{i}$ by an unknown stochastic factor is modeled following the Wiener process, where $W(t)\approx N(0,\Delta t)$, with a rate constant $\sigma$. Equation (2) is used to calculate the activation of the target protein $x_i$ by its upstream proteins as a linear combination of Boolean values of $B_k(w_{kj'})$ and the activity of protein $x_k$. In Eq. (2), $m^{'}$ is the total number of weights between $x_k$ and its upstream neighbors. Equation (3) calculates an inactivation of the target protein $x_i$ by interaction with its downstream proteins, where *m* is the total number of weights between $x_{i}$ and its downstream proteins. The *r* is the parameter of the Waller–Kraft operator, and $k_{u}$ and $k_{d}$ are rate constants.

### Model parameters and initial conditions

We choose the parameter values $k_{u}=0$ for the receptors and $k_d = 0$ for the protein without a downstream interacting counterpart. For all other proteins, we first set $k_u = k_{s}=1.0,$ and $r=0.75$ in the range of [0.5 1] of the Waller–Kraft operator, which is the range corresponding to “AND” logic of the Waller–Kraft operator.

We set the inactive states of protein to be zero ($x_i(0) = 0$). We assume that the receptors can be fully activated. We set $x_i(t_k)=1$, where *i* indicates receptor proteins that can be stimulated by the cell micro-environment, and $t_k$ represents the stimulation time. For the weights, we assume a uniform distribution between 0 and 1.

### Numerical calculation

Numerical calculations were performed using the Euler–Maruyama method, which is considered to be one of the simplest numerical approximations for stochastic differential equations. This method was derived from the Ito-Taylor expansion (supplementary for a brief derivation). If we truncate after the first order terms, we obtain the Euler–Maruyama method as follows:

4 $$\begin{aligned} X(t_{i+1})=X(t_{i})+f(X(t_{i}))\Delta t+g(X(t_{i}))\Delta W_{i}, \end{aligned}$$

where $\Delta t=t_{i+1}-t_{i}$, and $\Delta W_{i}=W(t_{i+1})-W(t_{i})$ for $i=0,\cdots ,n-1$ with the initial value $X(t_{0})=X_{0}$. The random variables $\Delta W_{i}$ are independent normally distributed random variables with mean 0 and standard deviation $\Delta t$.

## Results

### Model signaling pathway: simplified MAPK pathway

**Fig. 2:**
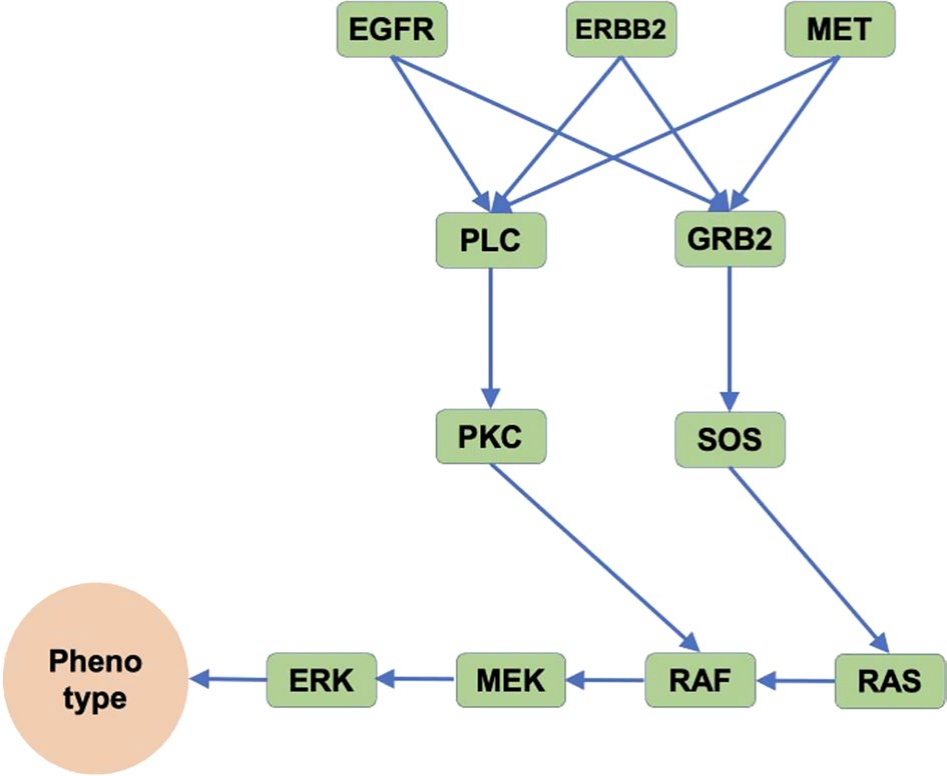
Schematic illustration of the simplified MAPK signaling pathway. Phenotype indicates one of the cell phenotypes regulated by ERK, such as cell proliferation, migration, and anti-apoptosis activity. The blue line represents the weight of protein–protein interactions

Utilizing the model and numerical simulation method described above in "Method" section, we simulated the activity of the simplified MAPK signaling pathway (Fig. 2), comprising 11 proteins and 14 interaction weights. In the model pathway, activated ERK is translocated across the nucleus membrane to regulate cell proliferation, migration, and anti-apoptosis effects [30]. These processes controlled by ERK are considered to be cell phenotypes in our MAPK signaling pathway. It is worth noting that all of the weights in the current model are assumed to be non-negative, representing positive interactions only. We chose this simplified MAPK pathway structure to test the current model, particularly the effect of the stochastic process on signaling transduction.

We take GRB2 as an example target protein to further explain the modeling process, as shown in Fig. 2. The GRB2, denoted by $$x_4$$, is activated by three immediately upstream proteins: EGFR ($$x_1$$), ERBB2 ($$x_2$$), and MET ($$x_3$$). GRB2 can stimulate only one downstream protein, SOS ($$x_6$$). EGFR ($$x_{1}$$) has two weights, $$w_{11}$$, and $$w_{12}$$; ERBB2 ($$x_{2}$$) has two weights, $$(w_{21}$$ and $$w_{22})$$; MET ($$x_{3}$$) has two weights, $$(w_{31}$$ and $$w_{32})$$; and GRB2 $$(x_{4})$$ has the only one weight, $$(w_{41})$$, for stimulating SOS. Therefore, the activities of upstream and downstream molecules of GRB2 can be expressed as follows:

$$\begin{aligned} {\text{upstream of GRB2:}} & \;\left( {r\min \left( {w_{{11}} ,w_{{12}} } \right) + \left( {1 - r} \right)\max \left( {w_{{11}} ,w_{{12}} } \right)} \right)x_{1}, \\ & + \;\left( {r\min \left( {w_{{21}} ,w_{{22}} } \right) + \left( {1 - r} \right)\max \left( {w_{{21}} ,w_{{22}} } \right)} \right)x_{2}, \\ & + \;\left( {r\min \left( {w_{{31}} ,w_{{32}} } \right) + \left( {1 - r} \right)\max \left( {w_{{31}} ,w_{{32}} } \right)} \right)x_{3}, \\ {\text{downstream of GRB2:}} & \;\left( {r\min \left( {w_{{41}} } \right) + \left( {1 - r} \right)\max \left( {w_{{41}} } \right)} \right)x_{4}. \\ \end{aligned}$$

The self-activity of GRB2 occurred by stochastic fluctuation can be expressed as $\sigma x_{4}(t)dW(t)$, where $W(t)\approx N(0,\Delta t)$.

### Single stimulus simulation

We first simulated the pathway upon receiving a single stimulus. We initially simulated the temporal evolution of proteins in the pathways without stochastic self-activity and then added the stochastic activity effect in subsequent simulations.

**Fig. 3:**
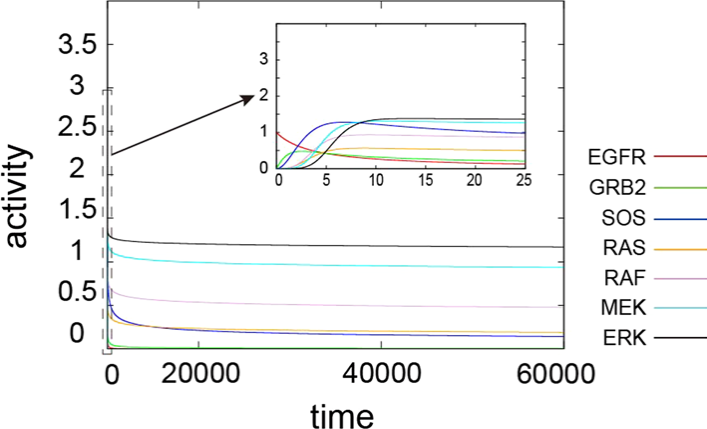
Temporal evolution of the MAPK signaling pathway without the stochastic self-activity from time step 0 to 60,000. In the early stage of the trajectories, each protein from time steps 0 to 25 (dotted rectangle range) is depicted as a zoomed-in inset figure. Red: EGFR, green: GRB2, blue: SOS, yellow: RAS, violet: RAF, cyan: MEK, black: ERK

We consider a case where the receptor EGFR is instantly activated by EGF initially ($x_i(0)$ = 1, where $x_i$ indicates EGFR). The activity of EGFR then decreases monotonically since no further EGF stimulation is applied. The activity of each downstream of EGFR increases sequentially according to the order of the MAPK signaling pathway (depicted in the dotted rectangle range in Fig. 3). The activity of each protein then sequentially decreases following the decrease in EGFR activity. The inactivation rates of all proteins were extremely slow (depicted as a solid rectangle in Fig. 3).

**Fig. 4:**
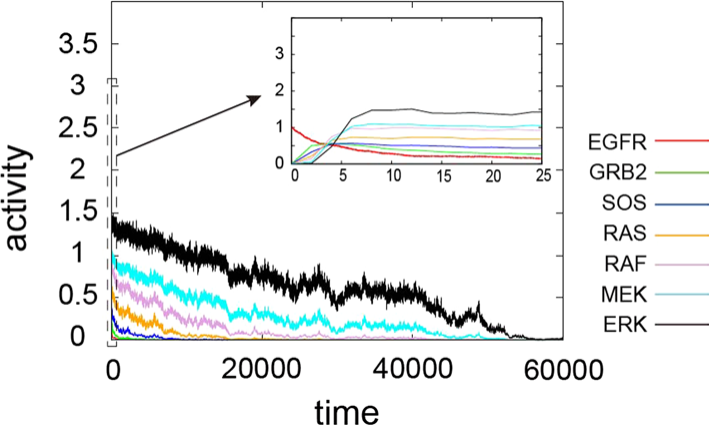
Trajectories for the activity of the MAPK signaling pathway with stochastic self-activity. Temporal evolution of protein activities from time step 0 to 60,000. In the early stage of the trajectories, each protein from 0 to 25 (dotted rectangle range) is depicted as a zoomed-in inset figure. Red: EGFR, green: GRB2, blue: SOS, yellow: RAS, violet: RAF, cyan: MEK, black: ERK

Figure 4 shows the trajectories of the activity of each protein in the MAPK signaling pathway when the stochastic self-activity was included. We set $\sigma = 1.0$ to solely investigate the influence of the stochastic process, *W*(*t*). Following EGFR activity, the proteins in the MAPK signaling pathway are sequentially activated (depicted in the solid rectangle range in Fig. 4). GRB2, downstream of EGFR, activates SOS, which in turn increases the activity of RAS, and so on down the pathway. The signal appeared to be amplified as it moved downstream in the pathway. Interestingly, the protein shifted to an inactive state (protein activity = 0) significantly faster than observed in the non-stochastic case (Fig. 4 vs. Fig. 3). We compared the decay time of all proteins in non-stochastic and stochastic cases (Additional file 1: Fig. S1). Since the protein activities did not decrease to near 0 in a non-stochastic case, we chose a specific time point, the time to reach 10% of the maximum value (*p*-value < 0.05, Student t-test). Although the random variables of the stochastic process *W*(*t*) were sufficiently small due to $\Delta t = 0.001$ and $W(t)\approx N(0,\Delta t)$, its impact on the MAPK pathway was nevertheless significant.

### Influence of weight in the MEK–ERK interaction

**Fig. 5:**
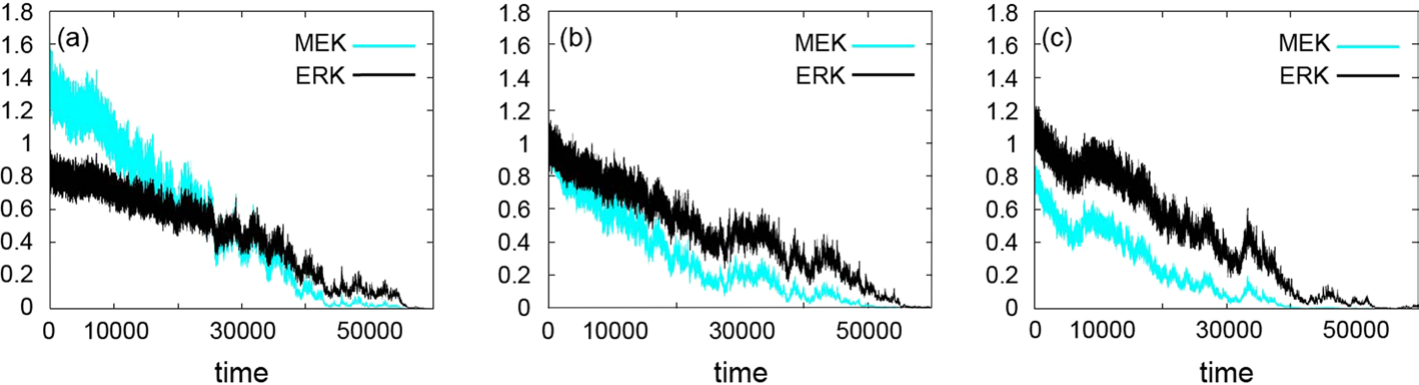
Trajectories for the activities of MEK (cyan) and ERK (black) from time step 0 to 60,000 assuming three different weights: 0.25 (**a**), 0.5 (**b**), 0.75 (**c**)

We next examined the influence of weight on protein activity. We focused on the weight of the MEK–ERK interaction to ignore the effect of a downstream protein affecting another protein. Figure 5 shows the trajectories of the activities of MEK and ERK. We set all weights to 0.5 except for the weight of the MEK–ERK interaction. We performed the simulation in response to a single stimulus assuming three different weights of MEK and ERK: 0.25, 0.5, and 0.75 (Fig. 5). In all cases, ERK activity was sustained over a longer period (Fig. 5), and there was a slower decrease in ERK (black) than MEK (cyan) activity. When the weight of the MEK–ERK interaction was smaller than the weights of other interactions, the initial activity of ERK was lower than that of MEK (Fig. 5a), although ERK activity caught up to reach the level of MEK activity at around time step 30,000. When the weight was greater than or equal to the weights of the other interactions, the ERK activity was maintained at a higher level for the entire period of the simulation (Fig. 5b, c).

### Multiple simulations

**Fig. 6:**
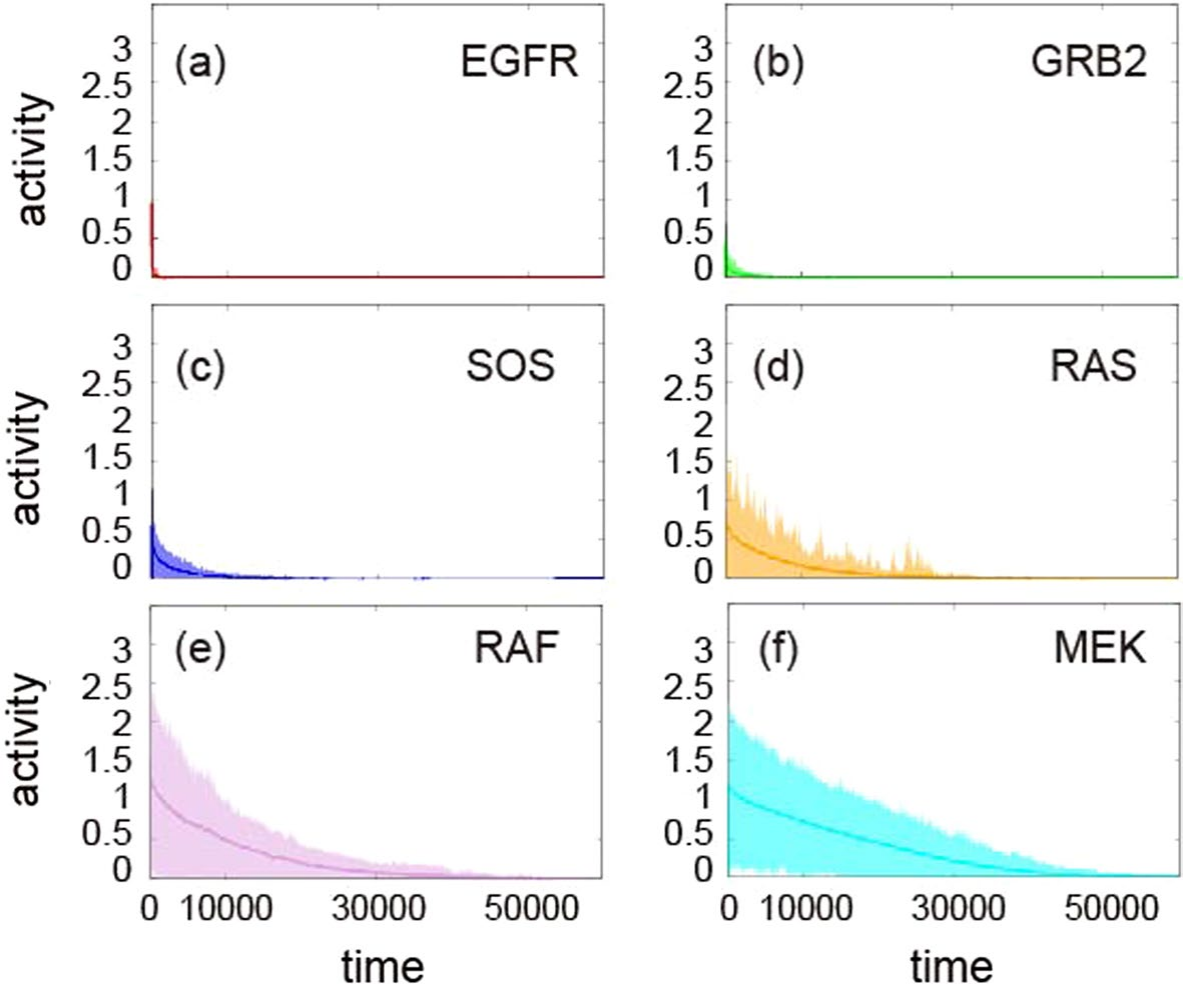
Temporal evolution of proteins of EGFR (**a**), GRB2 (**b**), SOS (**c**), RAS (**d**), RAF (**e**), and MEK (**f**) proteins. The solid line represents the average over the model simulation at each time step. The shaded area indicates the standard deviation

**Fig. 7:**
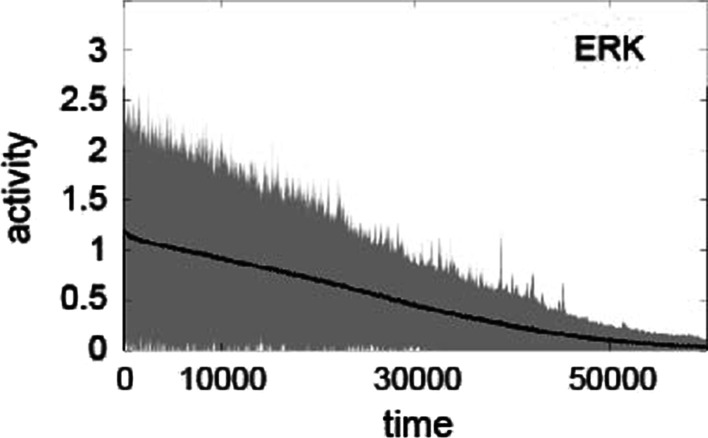
The trajectory of ERK from time 0 to 60000. The solid line shows the average of over 1000 simulations, and the shaded region indicates the standard deviation

To determine whether the stochastic effect on target protein activity significantly changes the overall behavior of the MAPK pathway, we conducted 1000 simulations for a single stimulus in which different random weights were assigned following a uniform distribution $w_{ij}^s \approx U[0,1]$, where $1 \le s \le 1000$ refers to each simulation trial. The temporal evolution of each protein appeared to be similar in all simulations (Fig. 6): all proteins were sequentially activated and then inactivated (converging to zero). We observed amplified signaling activity as the signal traveled downstream in the pathway (e.g., from EGFR to MEK), consistent with the observations for the single-simulation case (Fig. 4). Interestingly, the noise of protein activity also appeared to be increased when moving downstream in the pathway (RAF, MEK, ERK) compared to that upstream (EGFR, GRB2, SOS, and RAS) (Fig. 6, with a larger standard variation in (e) and (f), and Fig. 7.)

We also conducted additional simulations with different parameter sets of $k_{u},k_{d}$, and $\sigma$ (Additional file 1: Figs. S2–S8). Comparing with Figs. 6, 7, simulation results are qualitatively consistent. In particular, signaling amplification occurs in all parameter sets, although the amplitude of each protein activity rather changes with parameter sets. The smaller $k_d$ that regulates the downstream magnitude, the greater the target protein activation. The smaller the $k_u$, which governs the magnitude of the upstream, the smaller the activation of the target protein. A smaller stochastic factor ($\sigma$) delays inactivation.

**Fig. 8:**
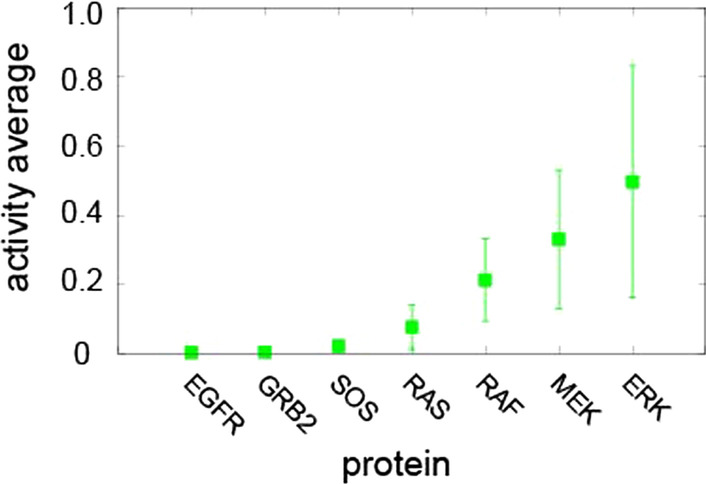
The overall activity of proteins from activation to the inactive state. Points represent the average over 1000 simulations, and error bars are the coefficient of variation

The parameter *r* is a coefficient of restriction in the Waller–Kraft operator. A value close to one imposes strong restrictions similar to the Boolean logic“AND”operation; on the other hand, a small value close to zero indicates less restriction, similar to a“OR”operation. In this study, we choose the “AND” logic of the Waller–Kraft operator to perform numerical simulations because the signal transduction between proteins simultaneously occurs within a sufficiently fast time ($10^{-9}$(ns)–$10^{-3}$(ms)). Even though we chose the “AND” logic, we compared the difference between “AND” and “OR” logic operators (Additional file 1: Figs. S9–12). It is worth noting that the simulation results with some large *r* values (e.g., $r \ge 0.75$, “AND” logic) did not show a significant difference. The results with “AND” logic are significantly different with some small *r* values (e.g., $r \le 0.25$, “OR” logic).

We next compared overall protein activities from stimulation to an inactive state. For each protein, we first calculated the area under the curve of the temporal profile. We then calculated the average over the areas and the coefficient of variation (Fig. 8). The coefficient of variation (CV) represents the ratio to the average value of the standard deviation; that is, if the sample standard deviation, *s*, and mean, $\mu$, then the CV is

$$\begin{aligned} \text {CV} = \frac{s}{\mu }. \end{aligned}$$

The actual value of the CV is dimensionless because it is independent of the unit in which the measurement was performed. It is useful to use coefficients of variation instead of standard deviations to compare data sets with different units or means. We observed an increasing tendency of overall protein activities from GRB2 to ERK. As the activity of the proteins increased, the range of the coefficient of variation due to the stochastic process also increased.

### Repeated stimulation of all receptors

**Fig. 9:**
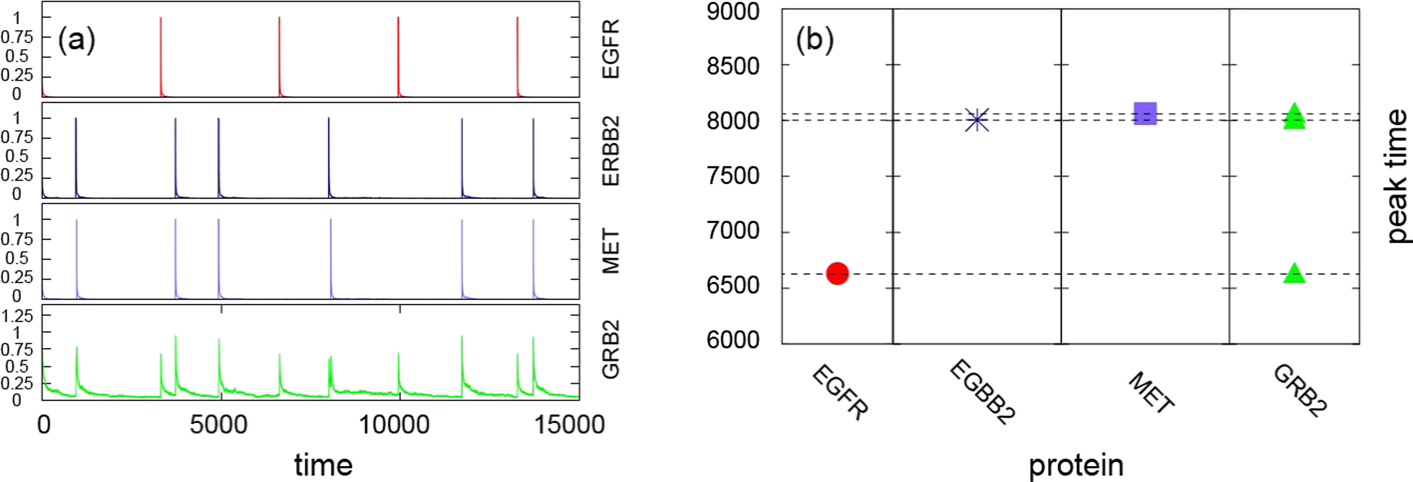
Temporal evolution of GRB2 activity. **a** the trajectory of GRB2 (bottom) reflects stimuli occurring at EGFR, ERBB2, and MET. **b** Timing of the highest activity (peak) of proteins EGFR, MET, ERBB2, and GRB2

Finally, we simulated the signaling pathway when the three receptors (EGFR, ERBB2, MET) were stimulated repeatedly. We conducted 1000 simulations while varying weights between proteins in the pathway. We set the receptors are fully activated initially ($x_{i}(0) = 1$, where $x_i$ stands for EGFR, ERBB2, or MET). After activation, the activity of the three proteins gradually decayed, returning to almost the inactive state (protein activity near zero). An additional stimulus was then applied to each receptor. Since the decrease rate of the activity differed for each receptor, the timing of additional activation also varied among the receptors (Fig. 9), resulting in different peak times for EGFR, ERBB2, and MET.

We observed various temporal evolution patterns of MAPK signaling pathway activity. From the representative temporal changes of the proteins, we observed that the activities of receptors increased quickly upon stimulation but also decreased rapidly unless additional stimulation was applied. The activity of GRB2 exhibited a time-delayed increase and a slower decrease compared with those of its upstream proteins (a representative example of the temporal evolution of protein activities is shown in Fig. 9a). Stimulation by additional EGFR activation induced a spike in GRB2 activity around the time step around 4000. Following additional stimulation by ERBB2 and MET around time step 4000 and 5000, GRB2 was again reactivated and subsequently decayed rather quickly (Fig. 9a). Interestingly, the activity of GRB2 exceeded one because ERBB2 and MET stimulated GRB2 almost simultaneously. To compare the timing of maximum activation, we compared the peak time (Fig. 9b) in the time interval [6000, 9000]. The peak time of the two receptors almost perfectly coincided with the peak time of GRB2.

**Fig. 10:**
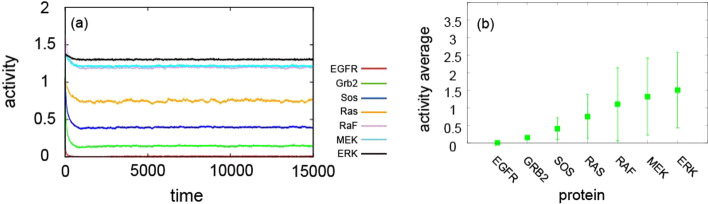
**a** Temporal evolution of average protein activities over 1000 model simulations in response to repeated stimulation on all the receptors. **b** The overall activity of proteins on a fixed time scale (from 0 to 15,000). Points represent the average over 1000 simulations, and error bars are the coefficient of variation

Interestingly, the average activities of MEK and ERK were maintained at a high level throughout the simulation time, even though the receptors were fully inactivated (Fig. 10a). Note that even though the stimulus repeatedly occurred, the average protein activity was maintained in a narrow range. Likewise, we compared overall protein activities over a fixed time scale, demonstrating an increasing tendency of overall protein activities from GRB2 to ERK. The range of coefficient of variation also appeared to be amplified, implying that the noise generated by the stochastic process propagates through the pathway when the signals travel downstream (Fig. 10b).

## Discussion

This paper presents a mathematical model of a cell signaling pathway based on the extended Boolean network model with a stochastic process. We applied the model to simulate a simplified MAPK pathway. Although several Boolean models have been developed to explain the signaling pathway [4, 6, 13], a mathematical model based on the extended Boolean model considering the weight by supplementing the drawback of a Boolean model has not been reported. Our model described the signaling pathway by taking into account protein scaffold formation, which is a natural phenomenon in which activated proteins are localized into polymer complexes through specific protein–protein interactions [20, 28, 29]. The scaffolding mechanism is known to promote efficient signal transduction and amplification [28] and employed in other pathways such as $\beta$-Arrestins and cAMP-dependent kinase’anchoring protein signaling [31]. Our model simulations of a single stimulus showed that the stochastic self-activity promoted a fast inactivation, although this stochastic effect could up-regulate each protein activity as well. It appears that the effect of stochastic down-regulation is more pronounced than stochastic up-regulation. With repeated stimulation on all of the receptors, we observed that the activated proteins promoted the activation of the protein in its immediate downstream neighboring protein, which in turn activated other proteins further downstream. The initial signaling was amplified and was sustained to activate downstream proteins as observed experimentally [28]. Interestingly, variation of protein activity due to stochastic sources was amplified as well (e.g., a large coefficient of variation of protein activity in ERK and MEK vs. a small one in EGFR in Fig. 8). A previous study of an ordinary differential equation model with stochastic updates also showed that noise is amplified in a MAPK/ERK pathway and this amplification helps as cell signal moves downstream. Importantly, the study showed that this noise amplification could improve information transmission [32].

The signaling pathway that we considered as an application of our proposed model is simplified pathway. In reality, cell signaling pathways are quite complex, typically including several positive or negative feedback loops [15, 16]. In the current study, we sought to understand the effect of stochastic factors on signaling amplification and decay. It is worth noting that having negative feedback in a pathway might modulate the over-activation or inactivation of a stimulated pathway. The model parameters such as interaction coefficient with upstream or downstream protein and weights between two proteins were not estimated with specific experimental data. Instead, we considered several different parameters to simulate the pathway and obtained qualitatively consistent results. In particular, one of the key predicted behaviors of the pathway, signaling amplification, was not dependent on parameters. The proposed model has not included several other factors of the signaling pathway such as protein-bonding speed, phosphorylation rate, and spatial interaction between proteins; however, adding more complexity does not guarantee a better understanding. For now, such experimental measurements of many proteins are not easily obtained. Therefore, while a more complex model may better represent a pathway in a relevant context, it could be burdened with additional model assumptions.

Our model was to numerically evidence the efficiency and suitability of the stochastic process and the extended Boolean network model. We chose our modeling approach as a starting point to understand the effect of stochastic factors on a cell signaling pathway. The model simulations suggested that the stochastic factor could modulate the decay rate of protein activity; a smaller stochastic coefficient resulted in slower decay. The results presented show the need to better understand the properties of stochastic factors in a cell signaling pathway. Detailed integration of the model with experimental data concerning stochastic factors could provide more quantitative predictions which can be tested in an experiment. Another extension includes the identification of interaction weights between proteins that modulate the signaling transduction [33].

## Supplementary Information

**Additional file 1.** Ito-Taylor expansion, Algorithms, Figure S1: The time to decay of all proteins in stochastic (left) and non-stochastic (right) cases, p-value < 0.05.  Figure S2: Average protein activity is taken from Figures 6-7 in the main text. Figure S3: Temporal evolution of average protein activity over 1000 simulations when the downstream coefficient is decreased to 50% compared to Fig. S2. Figure S4: Temporal evolution of average protein activity over 1000 simulations when the downstream coefficient is decreased to 10% compared to Fig. S2. Figure S5: Temporal evolution of average protein activity over 1000 simulations when the upstream coefficient is decreased to 50% compared to Fig. S2. Figure S6: Temporal evolution of average protein activity over 1000 simulations when the upstream coefficient is decreased to 10% compared to Fig. S2. Figure S7: Temporal evolution of average protein activity over 1000 simulations when the stochastic coefficient is decreased to 50% compared to Fig. S2. Figure S8: Temporal evolution of average protein activity over 1000 simulations when the stochastic coefficient is decreased to 10% compared to Fig. S2. Figure S9: Trajectories of the simplified MAPK pathway for each parameter r. Figure S10: Time to full inactivation for r = 1.0 and r = 0.75. Student t-test result, p-value = 0.9. Figure S11: Time to full inactivation for r = 1.0 and r = 0.0. Student t-test, p-value < 0.05. Figure S12: Time to full inactivation for r = 0.75 and r = 0.25. Student t-test, p-value < 0.05.

## Data Availability

All relevant data are within the paper.

## Abbreviations

MAPK: Mitogen-activated protein kinase
EGFR: Epidermal growth factor receptor
GRB2: Growth factor receptor-bound protein2
SOS: Son of sevenless
RAS: Renin angiotensin system
RAF: Rapidly accelerated fibrosarcoma
MEK: Mitogen-activated protein kinase kinase
ERK: Extracellular signal-regulated kinase

## References

[1] Kauffman SA (1969). Metabolic stability and epigenesis in randomly constructed genetic nets. J Theor Biol.

[2] Offermann B, et al. Boolean modeling reveals the necessity of transcriptional regulation for bistability in PC12 cell differentiation. Front Genet. 2016;7:44. 10.3389/fgene.2016.00044 PMC4830832 27148350

[3] Faure A (2006). Dynamical analysis of a generic Boolean model for the control of the mammalian cell cycle. Bioinformatics.

[4] Pandey S (2010). Boolean modeling of transcriptome data reveals novel modes of heterotrimeric G-protein action. Mol Syst Biol.

[5] Razzaq M (2018). Computational discovery of dynamic cell line specific Boolean networks from multiplex time-course data. PLoS Comput Biol.

[6] Eduati F (2020). Patient-specific logic models of signaling pathways from screenings on cancer biopsies to prioritize personalized combination therapies. Mol Syst Biol.

[7] Reka R (2017). A new discrete dynamic model of ABA-induced stomatal closure predicts key feedback loops. PLoS Biol.

[8] Montagud A (2022). Patient-specific Boolean models of signalling networks guide personalised treatments. Elife..

[9] Liang J (2012). Stochastic Boolean networks: An efficient approach to modeling gene regulatory networks. BMC Syst Biol.

[10] Zadrozny S, et al., An extended fuzzy Boolean model of information retrieval revisited. In: The 14th IEEE international conference on fuzzy systems, 2005. pp. 1020-1025.

[11] Waller WG (1979). A mathematical model of a weighted Boolean retrieval system. Inf Process Manag.

[12] Salton G (1983). Extended Boolean information retrieval. Commun ACM.

[13] van den Berg J (1997). Dynamic Boolean model. Stoch Process Appl.

[14] Tomida T (2015). Visualization of the spatial and temporal dynamics of MAPK signaling using fluorescence imaging techniques. J Physiol Sci.

[15] Nakakuki T (2010). Ligand-specific c-Fos expression emerges from the spatiotemporal control of ErbB network dynamics. Cell.

[16] Purvis JE (2013). Encoding and decoding cellular information through signaling dynamics. Cell.

[17] Santarpia L (2012). Targeting the MAPK-RAS-RAF signaling pathway in cancer therapy. Expert Opin Ther Targets.

[18] Dhillon AS (2007). MAP kinase signalling pathways in cancer. Oncogene.

[19] Chuderland D (2005). Protein–protein interactions in the regulation of the extracellular signal-regulated kinase. Mol Biotechnol.

[20] Witzel F (2012). How scaffolds shape MAPK signaling: what we know and opportunities for systems approaches. Front Physiol.

[21] Meister M (2013). Mitogen-activated protein (MAP) kinase scaffolding proteins: a recount. Int J Mol Sci.

[22] Shaw AS (2009). Scaffold proteins and immune-cell signalling. Nat Rev Immunol.

[23] Good MC (2011). Scaffold proteins: hubs for controlling the flow of cellular information. Science.

[24] Perkins TJ (2009). Strategies for cellular decision-making. Mol Syst Biol.

[25] Di Rienzo C (2014). Probing short-range protein Brownian motion in the cytoplasm of living cells. Nat Commun.

[26] Lafuerza LF (2011). Exact solution of a stochastic protein dynamics model with delayed degradation. Phys Rev E.

[27] Schultz D (2007). Molecular level stochastic model for competence cycles in Bacillus subtilis. Proc Natl Acad Sci.

[28] Lamson RE (2006). Dual role for membrane localization in yeast MAP kinase cascade activation and its contribution to signaling fidelity. Curr Biol.

[29] Morrison DK (2001). KSR: A MAPK scaffold of the Ras pathway?. J Cell Sci.

[30] Sebolt-Leopold JS (2006). Mechanisms of drug inhibition of signaling molecules. Nature.

[31] Mugabo Y (2018). Scaffold proteins: from coordinating signaling pathways to metabolic regulation. Endocrinology.

[32] Vazquez-Jimenez A (2019). On information extraction and decoding mechanisms improved by noisy amplification in signaling pathways. Sci Rep.

[33] Phizicky EM (1995). Protein–protein interactions: methods for detection and analysis. Microbiol Rev.

