# Supplementary material for: Mathematical model of the cell signaling pathway based on the extended Boolean network model with a stochastic process
Source: BMC Bioinformatics. 2022 Nov 30;23:515. doi: 10.1186/s12859-022-05077-z (PMC9710037; doi:10.1186/s12859-022-05077-z)
Supplement: Supplementary file 1 — Additional file 1. Ito-Taylor expansion, Algorithms, Figure S1: The time to decay of all proteins in stochastic (left) and non-stochastic (right) cases, p-value < 0.05. Figure S2: Average protein activity is taken from Figures 6-7 in the main text. Figure S3: Temporal evolution of average protein activity over 1000 simulations when the downstream coefficient is decreased to 50% compared to Fig. S2. Figure S4: Temporal evolution of average protein activity over 1000 simulations when the downstream coefficient is decreased to 10% compared to Fig. S2. Figure S5: Temporal evolution of average protein activity over 1000 simulations when the upstream coefficient is decreased to 50% compared to Fig. S2. Figure S6: Temporal evolution of average protein activity over 1000 simulations when the upstream coefficient is decreased to 10% compared to Fig. S2. Figure S7: Temporal evolution of average protein activity over 1000 simulations when the stochastic coefficient is decreased to 50% compared to Fig. S2. Figure S8: Temporal evolution of average protein activity over 1000 simulations when the stochastic coefficient is decreased to 10% compared to Fig. S2. Figure S9: Trajectories of the simplified MAPK pathway for each parameter r. Figure S10: Time to full inactivation for r = 1.0 and r = 0.75. Student t-test result, p-value = 0.9. Figure S11: Time to full inactivation for r = 1.0 and r = 0.0. Student t-test, p-value < 0.05. Figure S12: Time to full inactivation for r = 0.75 and r = 0.25. Student t-test, p-value < 0.05. [file 12859_2022_5077_MOESM1_ESM.pdf]

Mathematical model of the cell signaling pathway based on the extended Boolean network model with a stochastic process: Supplementary materials

Minsoo Kim and Eunjung Kim
Natural Product Informatics Research Center, Korea Institute of Science and Technology, Gangneung, Gangwon-do, Republic of Korea.

Ito-Taylor expansion

We briefly explain how we derived the numerical integration scheme for our stochastic differential equation from Ito-Taylor expansion [1].

First, we call a stochastic differential equation (SDE):

$$dX(t) = f(X(t))dt + g(X(t))dW(t), \tag{1}$$

where f and g are sufficiently smooth. Let F be the continuously differentiable function, then from Ito's lemma, we have

$$dF[X(t)] = f[X(t)]\frac{\partial F(X(t))}{\partial X} + \frac{1}{2}g^2[X(t)]\frac{\partial^2 F(X(t))}{\partial X^2}dt \tag{2}$$

$$+ g[X(t)]\frac{\partial F(X(t))}{\partial X}dW(t). \tag{3}$$

Next, we define operators as follows:

$$L^0 \equiv f[X(t)]\frac{\partial}{\partial X} + \frac{1}{2}g^2[X(t)]\frac{\partial^2}{\partial X^2}, \quad L^1 \equiv g[X(t)]\frac{\partial}{\partial X}. \tag{4}$$

Then, Eq. (2) can be rewritten as follow:

$$dF[X(t)] = L^0 F[X(t)]dt + L^1 F[X(t)]dW(t). \tag{5}$$

Taking integral, Eq. (5) is derived as:

$$X(t) = X_{t_0} + f[X(t_0)]\int_{t_0}^{t} d\tau_1 + g[X(t_0)]\int_{t_0}^{t} dW(\tau_1) + \mathcal{R}, \tag{6}$$

where $\mathcal{R}$ is the remaining terms which include the double integral terms:

$$\mathcal{R} \equiv \int_{t_0}^{t}\int_{t_0}^{\tau_1} L^0 f[X(\tau_2)]d\tau_2 d\tau_1 + \int_{t_0}^{t}\int_{t_0}^{\tau_1} L^1 f[X(\tau_2)]dW(\tau_2)d\tau_1$$
$$+ \int_{t_0}^{t}\int_{t_0}^{\tau_1} L^0 g[X(\tau_2)]d\tau_2 dW(\tau_1) + \int_{t_0}^{t}\int_{t_0}^{\tau_1} L^1 g[X(\tau_2)]dW(\tau_2)dW(\tau_1). \tag{7}$$

Selecting Eq. (4), we obtain

$$\begin{aligned} X(t) =& X_{t_0} + f[X(t_0)]\int_{t_0}^{t} d\tau_1 + g[X(t_0)]\int_{t_0}^{t} dW(\tau_1) \\ &+ g[X(t_0)]g^{'}[X(t_0)]\int_{t_0}^{t}\int_{t_0}^{\tau_1} dW(\tau_2)dW(\tau_1) + \tilde{\mathcal{R}}, \end{aligned} \tag{8}$$

where new remainder $\tilde{\mathcal{R}}$. Note that the double integral is evaluated to be

$$\int_{t_0}^{t}\int_{t_0}^{\tau_1} dW(\tau_2)dW(\tau_1) = \frac{1}{2}[W(t) - W(t_0)]^2 - \frac{1}{2}(t - t_0). \tag{9}$$

Substituting Eq. (9) into Eq. (8), we can produce the numerical integration scheme for the SDE from Ito-Taylor expansion with a time discretization $0 = t_0 < t_1 < \cdots < t_N = T$ of a time interval $[0, T]$ as follows:

$$\begin{aligned} X(t_{i+1}) =& X(t_i) + f(X(t_i))\triangle t + g(X(t_i))\triangle W_i \\ &+ \frac{1}{2} g(X(t_i))g^{'}(X(t_i))[(\triangle W_i)^2 - \triangle t] + \tilde{\mathcal{R}}, \end{aligned} \tag{10}$$

where $\triangle t = t_{i+1} - t_i$ and $\triangle W_i = W_{i+1} - W_i$ for $i = 0, 1, 2, \cdots, N-1$ with the initial condition $X(t_0) = X_0$. The random variable $\triangle W_i$ are independent $N(0, \triangle t)$ normally distributed random variables.

Algorithms

To explain the algorithms, we take an example of GRB2 and GRB2 upstream proteins such as EGFR, ERBB2, and MET (Fig.2). First, we calculate each Boolean value of EGFR, ERBB2, and MET based on a Boolean logic function (defined as **void** Boolean_gate_X).

```
# Function of X (X: EGFR, ERBB2, MET)
void Boolean_gate_X(···){
    int j;
    double w[num_weight];        # Numbers of weight of X
    w[0]=weight[weight1];
    w[1]=weight[weight2];
    double max=w[0];
    double min=w[0];
    for(j=0; j<num_weight; j++){
        if(w[j]<min){min=w[j];}
        if(max>w[j]){max=w[j];}}
    X_boo->boolean_value=r*min + (1.0-r)*max;
    downstream[order]=X_boo->boolean_value*protein[order];
```

Second, we estimate upstream flowing into GRB2 using upstream function (defined as **void** upstream_gate_grb2).

```
# Upstream function of GRB2
void upstream_gate_grb2(···){
 int j;
 double p[num_protein]; # protein flowing into GRB2
 double w[num_protein]; # weight flowing into GRB2
 upstream[order]=0.0;
 p[0]=protein[protein1];
 p[1]=protein[protein2];
 p[2]=protein[protein3];
 w[0]=egfr_boo->boolean_value;
 w[1]=erbb2_boo->boolean_value;
 w[2]=met_boo->boolean_value;
 for(j=0; j<num_protein; j++){
 upstream[order]+=p[j]*w[j];}}
```

Finally, the activity of GRB2 is calculated by a Boolean logic function (defined as **void** Boolean_gate_grb2) with the stochastic process.

```
# Function of GRB2
void Boolean_gate_grb2(···){
 double max=weight[weight1];
 double min=weight[weight1];
 grb2_boo->boolean_value=r*min+(1.0-r)*max;
 downstream[order]=grb2_boo->boolean_value*protein[order];
 new_protein[order]=protein[order]
 + ((kw*upstream-kp*downstream*protein[order])*dt
 + protein[order]*sigma*stochastic);}
```

The above functions are called repeatedly during simulation time.

```
for(k=0; k<=simulation time; k++){···
 for (i=0; i<Number of Protein; i++){···
  if(i==GRB2 order){
  double wiener=N(0,dt); #Normal distribution
  # downstream flowing out from X
  Boolean_gate_EGFR(protein, weight, downstream, X order,
  weight1 order, weight2 order, number of weight, X_boo);
  # Upstreams flowing into GRB2
  upstream_gate_grb2(protein, weight, upstream, i,
  GRB2 order, EGFR order, ERBB2 order, MET order,
  egfr_boo, erbb2_boo, met_boo, number of upstream);
```

```
# Calculation of Grb2 activity
Boolean_gate_grb2(protein ,weight ,downstream ,new_protein ,
upstream , i , weight order , grb2_boo , wiener);}
···}}
```

The effect of stochastic factors on protein inactivation

We compared inactivation time for all proteins in non-stochastic and stochastic cases (See Figures 3-7). Because the protein activities did not decrease to 0 in a non-stochastic case, we compare the time to decrease to a certain value (e.g., 10% of the maximum). The protein inactivation in the non-stochastic case is significantly delayed compared to the inactivation in the stochastic case.

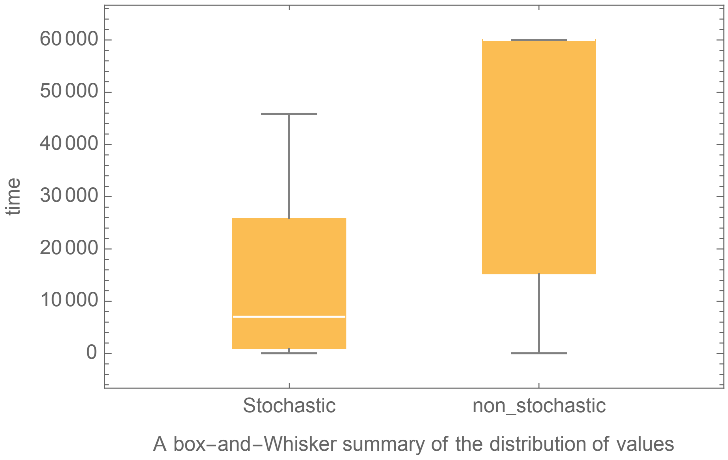

Figure S1: The time to decay of all proteins in stochastic (left) and non-stochastic (right) cases. p-value < 0.05.

Effect of model parameters

We performed additional simulations with different sets of parameter sets of k_u, k_d, σ, and r. First, we present an average protein activity over 1000 simulations when the three parameters are set to be one (See Figures 6-7 and Figure S2).

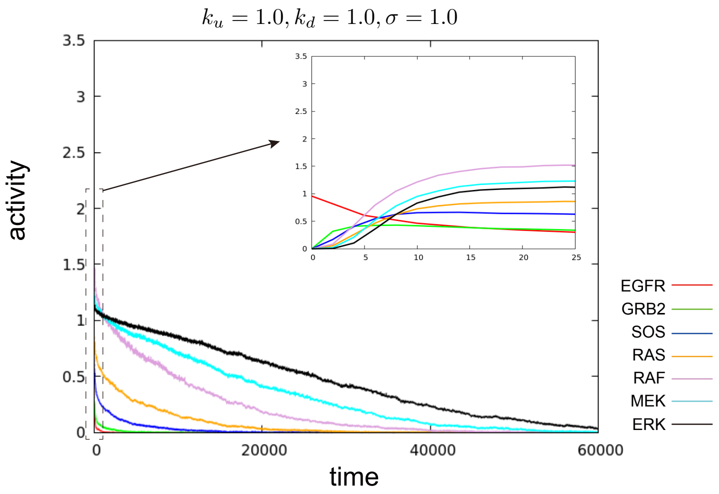

Figure S2: Average protein activity is taken from Figures 6-7 in the main text.

Effect of parameters k_d and k_u

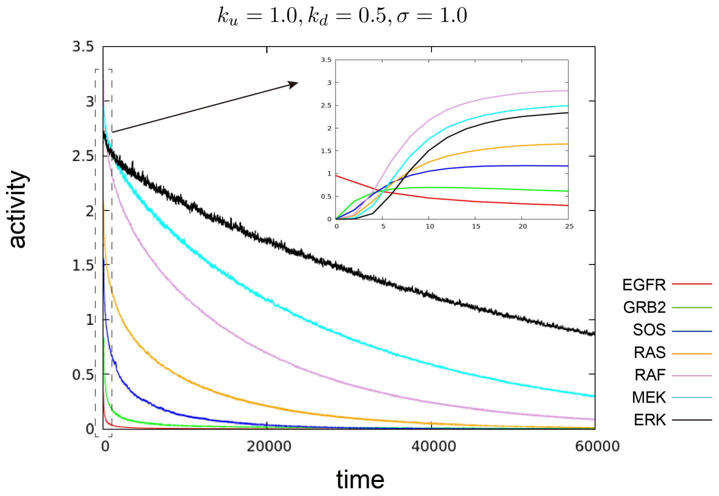

Figure S3: Temporal evolution of average protein activity over 1000 simulations when the downstream coefficient is decreased to 50% compared to Fig. S2.

To compare the effect of the downstream coefficient (k_d) on the signaling pathway, we considered two cases of $k_d = 0.5$ (Fig. S3) and $k_d = 0.1$ (Fig. S4). A decreased downstream coefficient makes the magnitude of each target protein activation larger. Although the signaling magnitude is different from Fig. S2, the order of protein amplification is maintained. In other words, the protein activity is sequentially amplified and sustained longer as

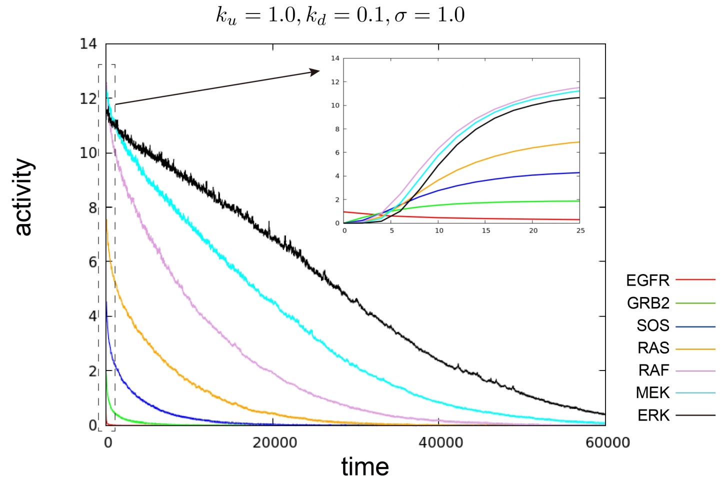

Figure S4: Temporal evolution of average protein activity over 1000 simulations when the downstream coefficient is decreased to 10% compared to Fig. S2.

the signal travels downstream. This key model prediction is not affected by the parameter change.

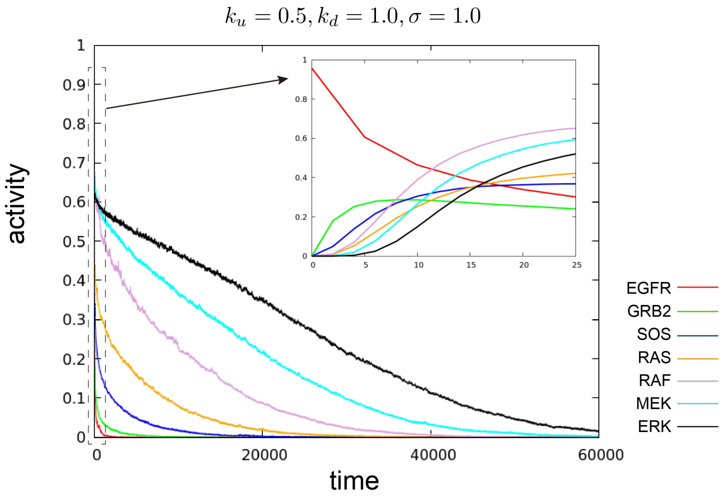

Figure S5: Temporal evolution of average protein activity over 1000 simulations when the upstream coefficient is decreased to 50% compared to Fig. S2.

Similarly, we performed 1000 simulations with a different upstream coefficient k_u (=0.5,0.1) in Fig. S5-Fig. S6. The upstream coefficient (k_u) affects the magnitude of the protein activation. A smaller coefficient makes the target protein activity smaller since an activation rate by an upstream protein

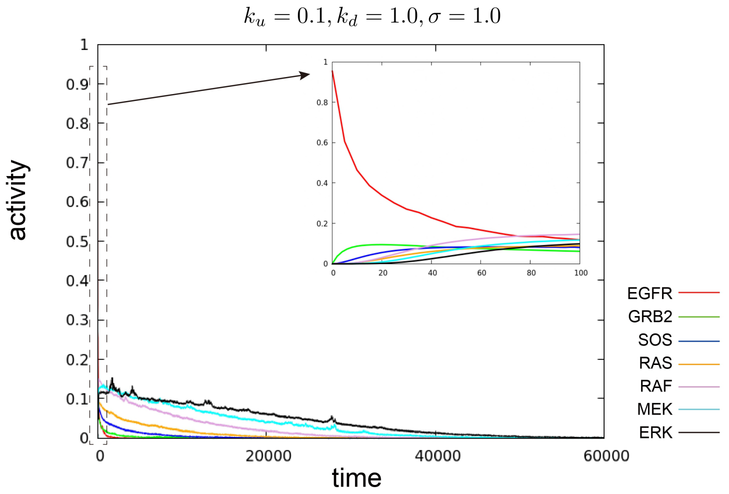

Figure S6: Temporal evolution of average protein activity over 1000 simulations when the upstream coefficient is decreased to 10% compared to Fig. S2.

of the target protein is smaller. The changes in the upstream coefficient did not affect the decay of protein activation nor the amplification of protein activity.

Effect of parameter σ

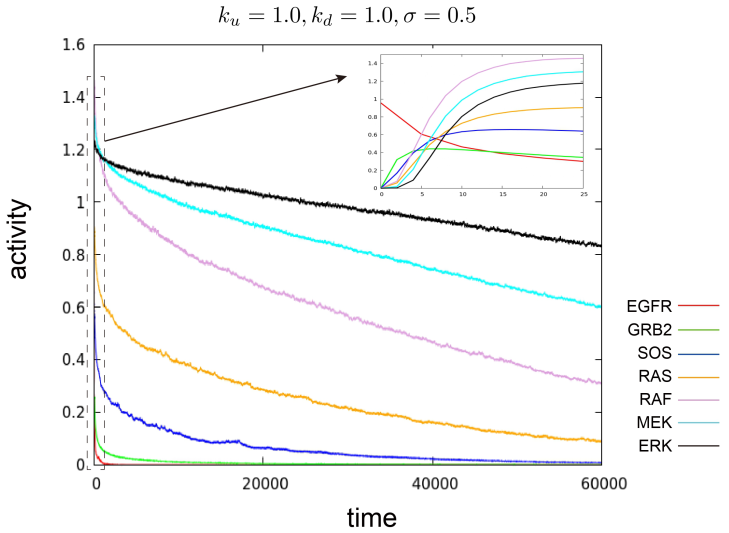

Figure S7: Temporal evolution of average protein activity over 1000 simulations when the stochastic coefficient is decreased to 50% compared to Fig. S2.

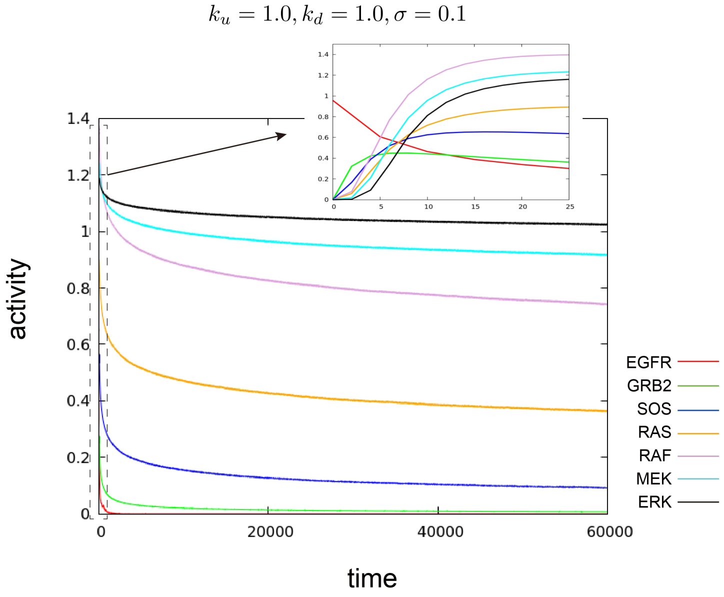

Figure S8: Temporal evolution of average protein activity over 1000 simulations when the stochastic coefficient is decreased to 10% compared to Fig. S2.

We considered two different stochastic coefficients $\sigma = 0.5, 0.1$. A smaller stochastic coefficient did not affect the magnitude of protein activation but delayed the protein inactivation (Fig. S7 - Fig. S8).

Effect of parameter r

To evaluate the effect of the parameter r, we considered four different values of r ($r = 0.0, 0.25, 0.75$ and $r = 1.0$). Figure S9 illustrates the temporal evolution of all proteins for each parameter r. Suppose the r value is one in the Boolean function, a minimum weight among the weights between the target protein and upstream proteins will be selected. Thus, a little activation of a target protein is expected by upstream proteins. Similarly, a minimum weight between a target and downstream proteins is selected, leading to a slower decay of a target protein. To quantify the effect of the parameter r on the signaling transduction, we calculated the time to almost full inactivation (e.g., a time point of each protein to reach less than 0.000001). We compare the time to this full inactivation of all proteins. The full inactivation time assuming $r = 1.0$ is not statistically different from the time assuming $r = 0.75$ (Fig. S10, Fig. S9a vs Fig. S9b). However, the time is significantly different from $r = 0.0$ (Fig. S11, Fig. S9a vs Fig. S9d). Lastly, the inactivation time assuming $r = 0.75$ is significantly different from $r = 0.25$ (Fig. S12, Fig. S9b

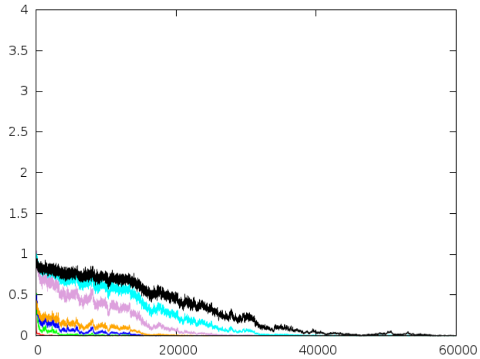

(a) Trajectory for $r = 1.0$

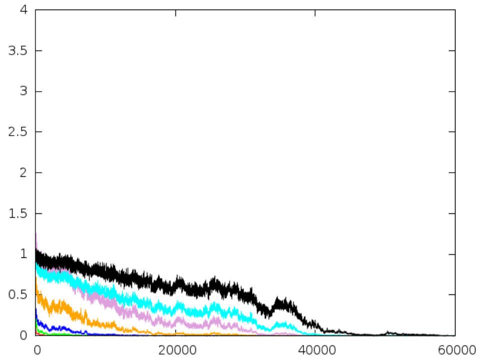

(b) Trajectory for $r = 0.75$

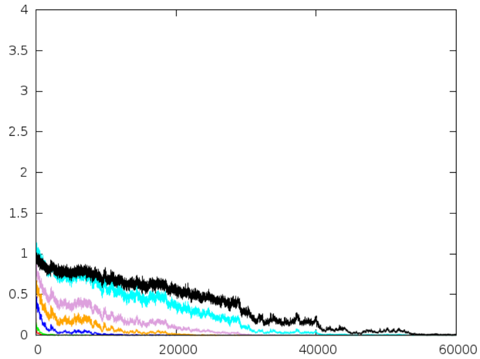

(c) Trajectory for $r = 0.25$

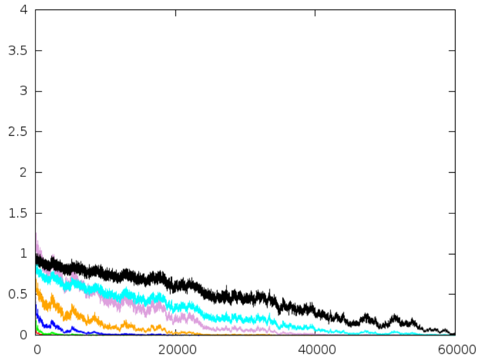

(d) Trajectory for $r = 0.0$

Figure S9: Trajectories of the simplified MAPK pathway for each parameter r.

vs Fig. S9c).

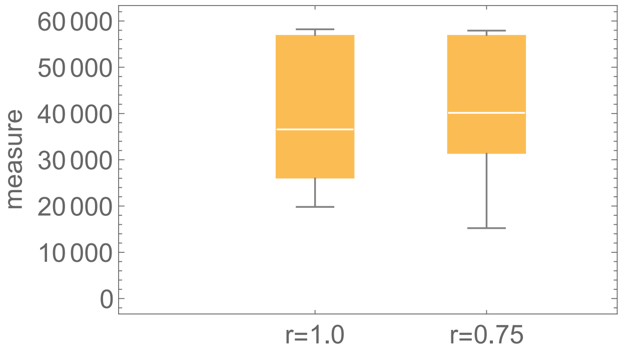

Figure S10: Time to full inactivation for $r = 1.0$ and $r = 0.75$. Student t-test result, p-value is 0.9.

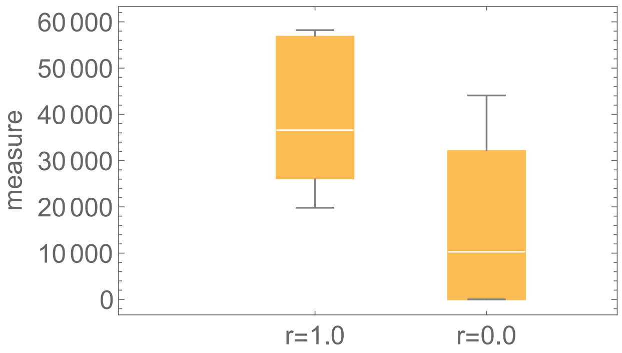

Figure S11: Time to full inactivation for $r = 1.0$ and $r = 0.0$. Student t-test, p-value < 0.05.

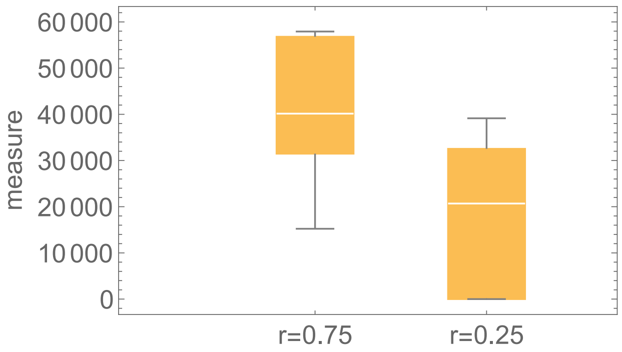

Figure S12: Time to full inactivation for $r = 0.75$ and $r = 0.25$. Student t-test, p-value < 0.05.

References

[1] Kloeden, P. E., et al., Stochastic Taylor Expansions. In Numerical Solution of Stochastic Differential Equations, Springer, Berlin, Heidelberg, 161-226, 1992.
